# Grapevine double cropping: a magic technology

**DOI:** 10.3389/fpls.2023.1173985

**Published:** 2023-04-14

**Authors:** Guilong Lu, Kai Zhang, Youxiong Que, Yanfeng Li

**Affiliations:** ^1^ Institute of Vegetables, Tibet Academy of Agricultural and Animal Husbandry Sciences, Lhasa, China; ^2^ Key Laboratory of Sugarcane Biology and Genetic Breeding, Ministry of Agriculture and Rural Affairs, Fujian Agriculture and Forestry University, Fuzhou, China

**Keywords:** grapevine, double cropping, flower bud differentiation, challenge, prospect

## Abstract

Grapevine is one of the most important fruit trees in the world, but it is often threatened by various biotic and abiotic stresses in production, resulting in decreased yield and quality. Grapevine double cropping in one year is a kind of preparatory and artificial control technology, which can not only save the loss of natural disasters, but also plays an important role in staggering the peak to market, thus increasing yield and improving the quality of grape fruit. This perspective provides a concise discussion of the physiological basis, the main determinants, and their impacts on yield and fruit quality of grapevine double cropping. We also highlight the current challenges around this theme and prospect its application in the future.

## Introduction

Grapevine (*Vitis vinifera* L.) is one of the earliest fruit trees domesticated and cultivated by human beings. It originated from contiguous regions of Eurasia and North America, and began to be cultivated between the Caspian Sea and Black Sea in Asia Minor and its south coast about 6,000 ~ 8,000 years ago, which gradually expanded to all over the world with cultural and economic exchanges (Hirst, 2021). It is now one of the most widely cultivated and economically valuable crops in the world. At present, there are more than 10,000 grape varieties, including about 3,000 cultivated, mostly with wine production, fresh food, dry, juice and other types. The cultivated area is about 7 million hectares, and the annual gross production value is more than 70 billion dollars (FAO, 2021).

During grape production, it is often subjected to abiotic and biotic stresses such as typhoon, hail, cold damage, drought, pest and diseases, resulting in yield reduction or even total crop failure in some areas (Louime et al., 2010). Due to the reason that some varieties also blossom and bear fruit after the disaster, the phenomenon did not attract enough attention at that time, but due to the lack of effective recovery management measures, the yield and quality of grape were seriously reduced. Until the 1930s, Баширов and Сушков from the former Soviet Union successively produced the second fruits from summer buds and winter buds at the same year. Since then, successful cases in India, Israel, China, Japan, Thailand and other countries have been reported continuously (Lu and Tudan, 2018), and in Lhasa (altitude 3,650 m), the roof of the world, the second fruit bearing has even been achieved in the facility grapes (Lu, 2019).

**Figure 1:**
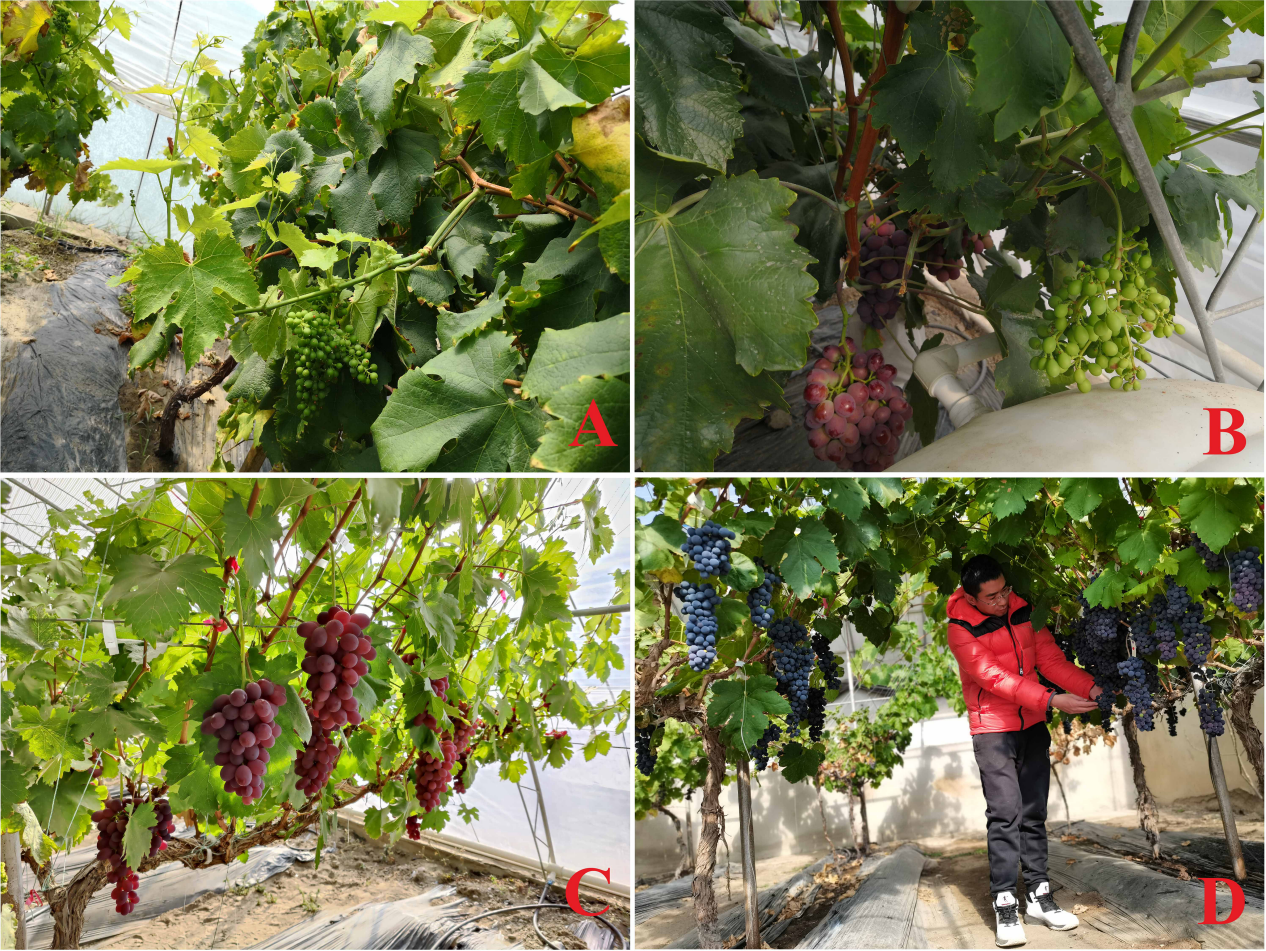
The second grape fruits of facility cultivation in Lhasa. **(A)** ‘Muscat Hamburg’ summer bud; **(B)** ‘Red Globe’ summer bud; **(C)** ‘Red Balado’ winter bud; **(D)** ‘Summer black’ winter bud.

In the long-term exploration, the new cultivation and management modes have gradually been shaped for grapevine double cropping: (1) two-crop-a-year grape cultivation that two crops are overlapped at some time, that is the growth periods of the first fruit and the second fruit partially overlap, but the maturity periods are staggered; (2) two-crop-a-year grape cultivation that two crops are not overlapped, in which the first and the second fruit will bear separately, and the growth periods will not overlap. Surprisingly, no matter in the field or facility cultivation, the second fruit can be produced by using summer buds or forcing winter buds to germinate according to local conditions, which has been confirmed on ‘Cabernet Sauvignon’, ‘Pinot Noir’, ‘Syrah’, ‘Muscat Hamburg’, ‘Red Balado’ and ‘Summer Black’ (**Figure 1**) (Gu et al., 2012; Bai et al., 2015; Guo et al., 2016; Junior et al., 2017; Lu, 2019; Poni et al., 2020).

The newly developed technology of grapevine double cropping is a standby technology and artificial control technology, which can not only reduce natural disaster losses, but also regulate the production period, ensure the annual supply of fresh fruit, and has great potential for development in terms of increasing production, income and efficiency. The aim of this perspective is to provide the latest overview on the research of grapevine double cropping, analyze the current challenges, and especially emphasize its development potential as a reserve technology.

## Physiological basis of grapevine double cropping

**Figure 2:**
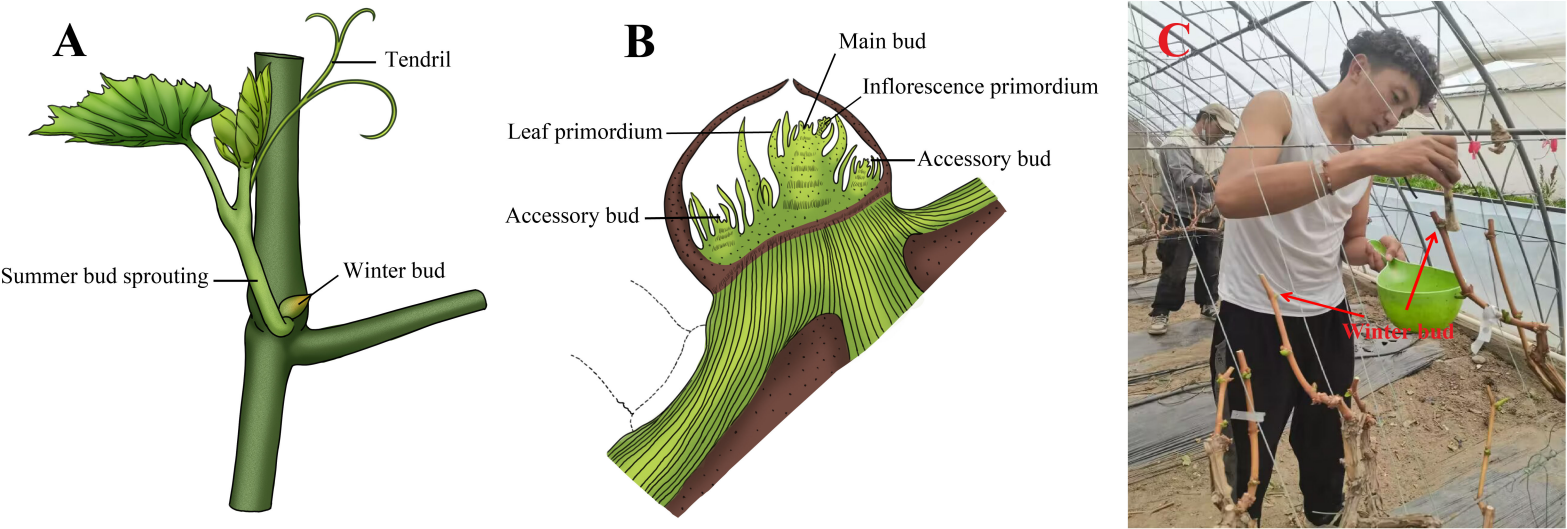
Grapevine growth diagram **(A)**, winter bud structure profile **(B)** and pruning after the harvesting of primary fruit and application of bud-breaking chemicals near the top first and second winter buds **(C)**.

Grapevine double cropping, that is, using the characteristics of grape summer buds that can be sprouted and flowered many times in a year, and the physiological differentiation feature of flower buds in winter buds that can be completed in the same year, combined with certain production measures to promote the sprouting of summer buds or winter buds formed in the same year to form the second fruit (Bai et al., 2015; Guo et al., 2016). In grapevine, summer buds are precocious, which grow side by side with the winter buds (**Figure 2A**). Generally, it can mature and germinate into accessory shoot about 20 d after leaf spreading. The ability for summer buds to form flowers is poor. The use of pinching and other measures can accelerate the differentiation process of flower bud, so that summer buds can germinate many times and form inflorescences. However, due to the short time of summer bud differentiation, the formed inflorescences are generally small (Vasconcelos et al., 2009). Grapevine winter buds are late maturing buds (**Figure 2B**). The differentiation generally starts around the flowering stage and is completed around the fruit maturity stage. The optimum temperature for flower bud formation is 20~30 °C, and the formation of flower primordium is most sensitive to temperature requirements, especially in the first three weeks (Srinivasan and Mullins, 1980). Generally, the winter buds near the lower part of the main branch are the first to differentiate, and the time, speed and integrity are affected by variety, temperature, light and other factors. Interestingly, they generally do not germinate in the year when they are formed, however they will only germinate and bear fruit when strongly stimulated by drought, pests and diseases, pruning, chemical treatment (**Figure 2C**) and other stress (Pellegrino et al., 2020; Poni et al., 2020; Martinez de Toda, 2021a).

In plants, the dormancy of winter buds is not the result of single hormone action, but closely related to the hormone balance (Or, 2009). Previous studies demonstrated that the formation of the second fruit of grapevine winter bud is closely related to the breaking of bud dormancy (Sudawan et al., 2016; Orrantia-Araujo et al., 2019), during which a series of physiological and biochemical changes will occur in the bud from dormancy to sprouting, such as the increase of endogenous hormones auxin (IAA), gibberellin (GA) and cytokinin (CTK), and the decrease of abscisic acid (ABA). Under the action of endogenous hormones, starch in buds is degraded into soluble sugar, which creates conditions for bud sprouting (He, 1999). During dormancy, late embryogenesis abundant (LEA) proteins, whose molecular structure contains a dehydrin region rich in Lys, are highly expressed in winter buds and can be induced by ABA (Or, 2009). ABA accumulates during the development of grapevine endoderm by inhibiting the activity of bud meristem, and its degradation is critical to dormancy release (Zheng et al., 2015; Zheng et al., 2018). Ethylene is suggested to participate in the degradation of ABA by regulating the expression of ABA signal regulator, thus promoting the breaking of dormancy (Oracz et al., 2008; Zheng et al., 2015). In addition, a higher proportion of zeatin-riboside/GA is conducive to promote the formation of more inflorescences (Guo et al., 2018), and studies have also confirmed that the flowering genes *LEAFY* (Li et al., 2011), *FT*, *TFL1A*, and *TFL1B* (Guo et al., 2018), and several miRNAs (*vv-miR160a*, *vv-miR171a*, *vv-miR159*, *vv-miR160a*, *vv-miR164c*, *vv-miR167c*) (Wang et al., 2011), are involved in the formation and development of grapevine flower buds.

## Determinants of grapevine double cropping application

### Variety

Grapevine is a warm-temperate crop that only begin to sprout and grow when the daily average temperate reaches 10°C in spring, and 10°C is thus generally referred to as grapevine biology zero. During the growing season, the accumulated temperature (i.e., active accumulated temperature) is the sum of the daily average temperature that is greater than or equal to 10°C during the year. Different grape types or varieties require different accumulated temperatures of more than 10 °C from sprouting to fruit maturity. In general, 2,100~2,500 °C for extreme early maturing grape varieties, 2,500~2,900 °C for early maturing grape varieties, 2,900~3,300 °C for medium maturing grape varieties, 3,300~3,700 °C for late maturing grape varieties, and more than 3,700 °C for extremely late maturing grape varieties (He, 1999). During the growth period of grape, if the accumulated temperature is insufficient for the second fruit, the ripening process cannot be successfully completed. Therefore, early and medium mature varieties with easy to form flower buds, good quality and short fruit bearing period should be developed as the first element for double cropping (Lu, 2019).

### Temperature

Regarding whether the open field conditions can achieve grapevine double cropping, the annual average temperature is most important. In the area with an average annual temperature of more than 20 °C, the two crops will not overlap for early and middle maturing varieties under natural conditions (Bai et al., 2015; Guo et al., 2016). While in the area with an average annual temperature of 12~20 °C, the model of two crops overlapped is suitable. Surprisingly, in facility cultivation, two modes of grape production, overlapped or unoverlapped, can be carried out even in cold areas (Ulanhot), where the annual average temperature is only about 5 °C through appropriate management measures (Guo et al., 2016).

### Pruning

During production, grape pruning can not only regulate the relationship between vegetative and reproductive growth, but also promotes the sprouting of summer buds and facilitates the winter buds to break dormancy (Poni et al., 2020; Martinez de Toda, 2021b). For summer buds, plucking 2~3 leaves above the inflorescence, and retaining 4~5 leaves from the accessory shoots sprouted at the top through repeated plucking, can promote the formation of the second inflorescence, while for winter buds, pinching 4~6 leaves on the inflorescence can promote the differentiation and concentration of flower buds, shorten the differentiation time, and improve the rate of flowering. For example, 4 or 6 leaves is profit for pinching on the inflorescence in ‘Summer Black’ (Zhu et al., 2020). It is shown that cutting and defoliating grapes after one fruit harvest can accelerate the flower bud differentiation of winter buds, and they even stimulate germinating and blossoming in some varieties (Gu et al., 2012; Martinez de Toda, 2021a). Besides, the node position and diameter of pruned branches are important for the formation of the second fruit of winter buds. Previous researches revealed that node 6 is suggested to be selected for ‘Summer Black’ (Zhu et al., 2020), middle nodes (8~10 nodes) for ‘Yatomi Rosa’ (Huang and Li, 2017), and high nodes (9~13 nodes) for ‘Kyoho’ for pruning (Li et al., 2013), which can not only ease the plant vigor, but also improve the bud sprouting rate and flower bud differentiation rate. In addition, the diameter of the remaining branches should be controlled above 0.8 cm, otherwise the nutrient accumulation of the bud is insufficient and the flower bud is difficult to differentiate (Fu et al., 2016).

### Chemical treatment

Under normal conditions, the grapes winter buds formed in that year do not germinate, and they must be treated with bud-breaking chemicals. The commonly used agents include nitrogen compounds, sulfur compounds, mineral oils and plant growth regulators, such as hydrogen cyanamide, lime nitrogen, and garlic extract (Srinivasan and Mullins, 1980; Leonei et al., 2015; Sudawan et al., 2016; Orrantia-Araujo et al., 2019). Among them, the dormancy breaking effect of monocyanine at a concentration of 2.5% was significant (Qiu et al., 2019), and the treated annual winter buds could generally sprout in 7~15 d, during which period the uniformity of winter bud sprouting were remarkably improved. Spraying an appropriate amount of chlormequat or paclobutrazol solution also plays an important role in accelerating the differentiation of winter buds and improving the rate of bearing branches of the second fruits (Lu and Tudan, 2018). In addition, due to the lack of low temperature stress, winter buds of grapevine planted in tropical or subtropical areas cannot pass the natural dormancy period, which means that they need bud-breaking chemicals to assist in sprouting (Sudawan et al., 2016).

### Cultivation management

The flower bud is formed by the combined effect of the accumulation of floral hormones and nutrients. If the nutrient accumulation is insufficient, the flower bud will mostly differentiate into tendrils (He, 1999; Monteiro et al., 2021). Therefore, strengthening the link between cultivation and management is important for the success of grapevine double cropping. For example, timely pruning shall be adopted to ensure ventilation and light transmission, while timely tendrils removing, flower and fruit thinning, and timely water and fertilizer supplying can ensure nutrient supply. Meanwhile, increasing microbial fertilizer can improve soil and living root, and timely cleaning and disinfecting the orchard are helpful for controlling and pest and disease. These comprehensive measures are not only beneficial to the healthy growth of grapevine and the differentiation of flower buds, but also create good conditions for the growth and thus improve the yield and quality of grape fruits (Morinaga, 2001; Pommer, 2006; Szabo and Shojania, 2019).

### Other

The differentiation process of grape winter buds is also affected by many factors, such as light level, drought stress, disease infection and pest stress. Furthermore, strong light and moderate drought stress are conducive to the maturity of winter buds (Vasconcelos et al., 2009; Pellegrino et al., 2020). When the scales of winter bud turn yellow, the scales edge is light brown, and the branches are semi-lignified, it may indicate that the differentiation is basically completed (Lu and Tudan, 2018). According to this feature and the characteristics of grape varieties, an appropriate cultivation mode of grapevine double cropping can be established.

## The impact on fruit quality and yield of grapevine double cropping

### Quality

Previous studies have shown that the fruit flavor and quality of the second fruit of grapevine is obviously better than that of the primary fruit, although the spike weight, single grain weight and size of the former are smaller than those of the latter (Ahmed et al., 2019; Qiu et al., 2019). Specifically, the content of soluble solids (Junior et al., 2017), flavonoids (Chen et al., 2017; Wang et al., 2022; Cheng et al., 2023), phenolic compounds (Xu et al., 2011; Cheng et al., 2019; Lu et al., 2022), volatile compounds (Chen et al., 2021; Lu et al., 2021), and tartaric acid and malic acid (Poni et al., 2020; Martinez de Toda, 2021a) in the second fruit are significantly increased, and compared to the primary fruit, the major components of flavonoids, phenolic compounds and volatile compounds were different in the second fruit. Besides, the growth period of second fruit grape with more beautiful color (Chen et al., 2017; Cheng et al., 2017; Ahmed et al., 2019; Cheng et al., 2023), is significantly shorter than that of the primary fruit (Koyama et al., 2020). This is mainly related to the large temperature difference between day and night, less rainfall, low water-heat coefficient before harvesting in the environment where the second fruit of grape grows, and the less occurrence of pests and diseases in this period, which is more conducive to the improvement of fruit quality (Cheng et al., 2017; Cheng et al., 2019; Chen et al., 2021; Wang et al., 2022).

### Yield

Grapevine double cropping can generally increase the yield by 10%~20% per year, but if the primary fruit yield is too high, it will increase the nutrient consumption of the tree body, which has a negative impact on the maturity and quality of the second fruit (Lu and Tudan, 2018). To ensure the stability of yield and quality of grapes harvested twice a year, the yield ratio of the second fruit to primary fruit should be controlled at 2:5~3:5 (Qiu et al., 2019). If the mode of two crops not overlapped are used, the trees should be left at least for more than 20 d after the first fruit harvest, and nutrition should be supplemented in time to better restore the tree vigor and improve the yield and quality of the second fruits (Lu and Tudan, 2018). In addition, it is of great significance to regulate the yield and marketing time of grape production reasonably in the primary and second fruits, which is also important to avoid the phenomenon of major or minor year, maintain the health of grape tree and its reasonable growth life.

## Challenges and prospects

### Challenge

The flower bud is the basis and key to grapevine double cropping. It is relatively simple to use the accessory shoots of summer buds to produce the second fruits, and repeated pinching is used to promote the formation of flower buds. However, due to the inability to uniformly control the flowering time, it frequently causes uneven fruit bearing period, ear size and quality. And the technology of using grape winter flower bud to germinate to form the second fruit is much more complicated. The unified regulation and management have the advantages of sprouting neatly, similar ear size, consistent maturity, good quality, beautiful color, and high price of winter grapes, which have good market competitive advantages. At present, winter buds are mainly used in production. However, the current research still faces challenges: (1) the hormone changes in the whole grape growth and development process and the regulation mechanism of flowering genes are not completely clear; (2) the side effects of the use of bud-breaking chemicals, as well as the more environmentally friendly and convenient method of breaking the winter bud sleep also need to be further studied; (3) the corresponding cultivation techniques of different climatic types and grape varieties still need further research and improvement; and (4) the specific effects of double cropping production on grape growth characteristics (including tree vigor, result life) and fruit quality also need to be further explored.

### Prospect

The edaphoclimatic conditions are essential to increase the competitiveness of plant productive system, and in this sense, global warming is partly conducive to the application of the two harvest one year for grapes. Moreover, the world population will exceed 9.7 billion by 2050 (United Nations, 2022), and the demand for grapes will further increase, while at the same time we also faced with the urgent dilemma of frequent global extreme climate, limited available land resources and continuous challenges of industrialization and urbanization. It is no doubt that, in order to guarantee/improve the yield and quality of grapes per unit, we should take precautions and constantly improve the reserve technology for two harvest one year, especially when we are in face of current and future unpredictable difficulties. In conclusion, grapevine double cropping has broad application prospects in future production, and we recommend that it should be reasonably applied and promoted according to the scientific laws of grape growth.

## Data availability statement

The original contributions presented in the study are included in the article/supplementary material. Further inquiries can be directed to the corresponding authors.

## Ethics statement

Written informed consent was obtained from the individual(s) for the publication of any identifiable images or data included in this article.

## Author contributions

GL: Methodology, Formal analysis, Software, Visualization, Writing - original draft. KZ: Visualization. YQ: Conceptualization, Methodology, Visualization, Supervision, Writing - review & editing. YL: Conceptualization, Funding acquisition, Supervision, Resources, Project administration. All authors contributed to manuscript revision, read, and approved the submitted version.

## References

[B1] Ahmed S. Roberto S. R. Shahab M. Colombo R. C. Silvestre J. P. Koyama R. . (2019). Proposal of double-cropping system for ‘BRS isis’ seedless grape grown in subtropical area. Sci. Hortic Amsterdam 251, 118–126. doi: 10.1016/j.scienta.2019.03.022

[B2] Bai X. Chen A. Cao M. Xie T. Zhang Y. Wen R. . (2015). “Key technique of two-crop-a-year grape cultural in sourthern of China,” in Abstracts of 2015 annual academic conference of Chinese horticultural society (Xiamen: Editorial office of Acta Horticulturae Sinica), 2605.

[B3] Chen W. Bai X. Cao M. Cheng G. Cao X. Guo R. . (2017). Dissecting the variations of ripening progression and flavonoid metabolism in grape berries grown under double cropping system. Front. Plant Sci. 8. doi: 10.3389/fpls.2017.01912 PMC5686318 29176986

[B4] Chen Y. Luo H. Lu M. Nong H. Bai Y. Lin L. . (2021). Aroma components analysis of summer black grape under two-crops-a-year cultivation. J. South. Agric. 52, 1343–1352. doi: 10.3969/j.issn.2095-1191.2021.05.025

[B5] Cheng G. Zhang J. Zhou S. Xie L. Zhang Y. Yang Y. . (2017). Difference in anthocyanin composition between winter and summer grape berries of ‘Cabernet sauvignon’ under two-crop-a-year cultivation. J. Fruit Sci. 34, 1125–1133. doi: 10.13925/j.cnki.gsxb.20170032

[B6] Cheng G. Zhou S. Liu J. Feng Q. Wei R. Yu H. . (2023). Widely targeted metabolomics provides new insights into the flavonoid metabolism in ‘Kyoho’ grapes under a two-crop-a-year cultivation system. Horticulturae 9, 154. doi: 10.3390/horticulturae9020154

[B7] Cheng G. Zhou S. Zhang J. Huang X. Bai X. Xie T. . (2019). Comparison of transcriptional expression patterns of phenols and carotenoids in ‘Kyoho’ grapes under a two-crop-a-year cultivation system. PloS One 14, e0210322. doi: 10.1371/journal.pone.0210322 30629640 PMC6328245

[B8] Food and Agriculture Organization of the United Nations (FAO) (2021) FAO statistical databases. Available at: http://www.fao.org/faostat/zh/#data/ (Accessed January 29, 2023).

[B9] Fu X. Liu X. He M. Chen T. Lei Y. (2016). Experimental study on the winter kyoho with two separate fruiting periods per year. South China Fruits 45, 135–137. doi: 10.13938/j.issn.1007-1431.20160129

[B10] Gu S. Jacobs S. D. Mccarthy B. S. Gohil H. L. (2012). Forcing vine regrowth and shifting fruit ripening in a warm region to enhance fruit quality in ‘Cabernet sauvignon’ grapevine (*Vitis vinifera* l.). J. Hortic. Sci. Biotech. 87, 287–292. doi: 10.1080/14620316.2012.11512866

[B11] Guo R. Wang B. Cheng G. Lin L. Cao X. Zhang Y. . (2016). Research advances in regionalization for two-crop-a-year grape cultivation in China. J. South. Agric. 47, 2091–2097. doi: 10.3969/j:issn.2095-1191.2016.12.2091

[B12] Guo R. Wang B. Lin L. Cheng G. Zhou S. Xie S. . (2018). Evolutionary, interaction and expression analysis of floral meristem identity genes in inflorescence induction of the second crop in two-crop-a-year grape culture system. J. Genet. 97, 439–451. doi: 10.1007/s12041-018-0929-5 29932064

[B13] He P. C. (1999). Grapeviology (Beijing: China Agriculture Press), 60.

[B14] Hirst K. K. (2021) Vitis vinifera: Origins of the domesticated grapevine. Available at: https://www.thoughtco.com/origins-of-the-domesticated-grape-169378.

[B15] Huang J. Li J. (2017). Effects of summer shearing at different nodes on stimulating winter bud germination and flower formation of grape. Northwest Hortic. 30, 47–48.

[B16] Junior M. J. P. Hernandes J. L. Bardin-Camparotto L. Blain G. C. (2017). Plant parameters and must composition of ‘Syrah’ grapevine cultivated under sequential summer and winter growing seasons. Bragantia 76, 345–351. doi: 10.1590/1678-4499.146

[B17] Koyama R. Borges W. F. S. Colombo R. C. Hussain I. Souza R. T. Roberto S. R. (2020). Phenology and yield of the hybrid seedless grape ‘BRS melodia’ grown in an annual double cropping system in a subtropical area. Horticulturae 6, 3. doi: 10.3390/horticulturae6010003

[B18] Leonei S. Tecchio M. A. Coser G. M. A. G. (2015). Dormancy breaking of fig tree with hydrogen cyanamide and garlic extract. Br. J. Appl. Sci. Technol. 10, 1–10. doi: 10.9734/BJAST/2015/18194

[B19] Li Z. Li J. Guo L. Zhang Y. Dong D. Cao M. (2011). Expression of *LEAFY* gene during flower bud differentiation of ‘Kyoho’ grapevine. Biotechnol. Bull. 22, 41–44. doi: 10.3969/j.issn.1009-0002.2011.01.010

[B20] Li H. Xie T. Cao M. Liu J. Chen G. Wen R. (2013). Effects of different prune section bits on winter fruit of one-year-two-harvest kyoho grape. Southwest China J. Agric. Sci. 26, 2170–2172. doi: 10.16213/j.cnki.scjas.2013.05.072

[B21] Louime C. Vasanthaiah H. K. Basha S. M. Lu J. (2010). Perspective of biotic and abiotic stress research in grapevines (*Vitis* sp.). Int. J. Fruit Sci. 10, 79–86. doi: 10.1080/15538361003676819

[B22] Lu G. (2019). Preliminary study on secondary results of Lhasa facility grape. Tibet J. Agric. Sci. 401, 36–39. doi: 10.3969/j.issn.1005-2925.2019.01.011

[B23] Lu H. Chen W. Wang Y. Bai X. Cheng G. Duan C. . (2021). Effect of the seasonal climatic variations on the accumulation of fruit volatiles in four grape varieties under the double cropping system. Front. Plant Sci. 12. doi: 10.3389/fpls.2021.809558 PMC8829325 35154206

[B24] Lu H. Chen W. Wang Y. Bai X. Cheng G. Duan C. . (2022). Effect of the seasonal climatic variations on the flavonoid accumulation in *Vitis vinifera* cvs. ‘Muscat hamburg’ and ‘Victoria’ grapes under the double cropping system. Foods 11, 48. doi: 10.3390/foods11010048 PMC8750161 35010174

[B25] Lu G. Tudan J. (2018). Research progress of the second fruiting of grape in China. J. Agric. 8, 68–72. doi: 10.11923/j.issn.2095-4050.cjas18080022

[B26] Martinez de Toda F. (2021a). Global warming allows two grape crops a year, with about two months apart in ripening dates and with very different grape composition -the forcing vine regrowth to obtain two crops a year. Vitis 60, 119–124. doi: 10.5073/VITIS.2021.60.119-124

[B27] Martinez de Toda F. (2021b). Grapevine double cropping: a reality, not a myth. IVES Tech. Rev. Vine Wine. doi: 10.20870/IVES-TR.2021.4572

[B28] Monteiro A. I. Malheiro A. C. Bacelar E. A. (2021). Morphology, physiology and analysis techniques of grapevine bud fruitfulness: a review. Agriculture 11, 127. doi: 10.3390/agriculture11020127

[B29] Morinaga K. (2001). “Grape production in Japan,” in Grape production in the Asia-pacific region. Eds. Papademetriou M. K. Dent F. J. (Bangkok: FAO Regional Office for Asia and the Pacific (RAP Publication 2000/13). Available at: https://www.fao.org/3/X6897E/x6897e07.htm.

[B30] Or E. (2009). “Grape bud dormancy release-the molecular aspect,” in Grapevine molecular physiology & biotechnology. Ed. Roubelakis-Angelakis K. A. (Dordrecht: Springer), 1–29. doi: 10.1007/978-90-481-2305-6_1

[B31] Oracz K. El-Maarouf-Bouteau H. Bogatek R. Corbineau F. Bailly C. (2008). Release of sunflower seed dormancy by cyanide: cross-talk with ethylene signalling pathway. J. Exp. Bot. 59, 2241–2251. doi: 10.1093/jxb/ern089 18448476 PMC2413275

[B32] Orrantia-Araujo M. A. Martínez-Téllez M. A. Corrales-Maldonado C. Rivera-Domínguez M. Vargas-Arispuro I. (2019). Changes in glutathione and glutathione disulfide content in dormant grapevine buds treated with garlic compound mix to break dormancy. Sci. Hortic Amsterdam 246, 407–410. doi: 10.1016/j.scienta.2018.10.064

[B33] Pellegrino A. Rogiers S. Deloire A. (2020). Grapevine latent bud dormancy and shoot development. IVES Tech. Rev. Vine Wine. doi: 10.20870/IVES-TR.2020.3420

[B34] Pommer C. V. (2006). Double cropping of table grapes in Brazil. Chronica Hortic. 46, 22–25.

[B35] Poni S. Gatti M. Tombesi S. Squeri C. Sabbatini P. Lavado N. . (2020). Double cropping in *Vitis vinifera* l. pinot noir: myth or reality? Agronomy 10, 799. doi: 10.3390/agronomy10060799

[B36] Qiu Z. Chen G. Qiu D. (2019). Pruning and dormancy breaking make two sustainable grape-cropping productions in a protected environment possible without overlap in a single year. PeerJ 7, e7412. doi: 10.7717/peerj.7412 31396448 PMC6681797

[B37] Srinivasan C. Mullins M. G. (1980). Effects of temperature and growth regulators on formation of anlagen, tendrils and inflorescences in *Vitis vinifera* l. Ann. Bot. London 145, 439–446. doi: 10.1093/oxfordjournals.aob.a085842

[B38] Sudawan B. Chang C. Chao H. Ku M. S. B. Yen Y. (2016). Hydrogen cyanamide breaks grapevine bud dormancy in the summer through transient activation of gene expression and accumulation of reactive oxygen and nitrogen species. BMC Plant Biol. 16, 202. doi: 10.1186/s12870-016-0889-y 27627883 PMC5024461

[B39] Szabo P. V. Shojania J. (2019). Growing grapes – managing the vineyard (New York: Nova Science Publishers). Available at: https://grapes.extension.org/growing-grapes-managing-the-vineyard/.

[B40] United Nations Department of Economic and Social Affairs (UNDESA) Population Division (2022). World population prospects 2022: Summary of results (New York: UNDESA). Available at: https://desapublications.un.org/publications/world-population-prospects-2022-summary-results.

[B41] Vasconcelos M. C. Greven M. Winefield C. S. Trought M. C. T. Raw V. (2009). The flowering process of *Vitis vinifera*: a review. Am. J. Enol. Viticult. 60, 411–434. doi: 10.5344/ajev.2009.60.4.411

[B42] Wang B. Qin F. Deng F. Luo H. Chen X. Cheng G. . (2022). Difference in flavonoid composition and content between summer and winter grape berries of shine Muscat under two-crop-a-year cultivation. Sci. Agric. Sin. 55, 4473–4486. doi: 10.3864/j.issn.0578-1752.2022.22.012

[B43] Wang C. Sun X. Fang J. Leng X. Li X. Mu X. (2011). Spatiotemporal expression of five microRNAs and their target genes during flower development of treated winter buds of grapevine in growing season. Acta Bot. Bor. Occid. Sin. 31, 2429–2436.

[B44] Xu C. Zhang Y. Zhu L. Huang Y. Lu J. (2011). Influence of growing season on phenolic compounds and antioxidant properties of grape berries from vines grown in subtropical climate. J. Agr. Food Chem. 59, 1078–1086. doi: 10.1021/jf104157z 21235208

[B45] Zheng C. Acheampong A. K. Shi Z. Mugzech A. Halaly-Basha T. Shaya F. . (2018). Abscisic acid catabolism enhances dormancy release of grapevine buds. Plant Cell Environ. 41, 2490–2403. doi: 10.1111/pce.13371 29907961

[B46] Zheng C. Halaly T. Acheampong A. K. Takebayashi Y. Jikumaru Y. Kamiya Y. . (2015). Abscisic acid (ABA) regulates grape bud dormancy, and dormancy release stimuli may act through modification of ABA metabolism. J. Exp. Bot. 66, 1527–1542. doi: 10.1093/jxb/eru519 25560179 PMC4339608

[B47] Zhu W. Lin L. Xie S. Han J. Cao M. Guo R. . (2020). Effects of pinching on flower differentiation in winter buds at different nodes of ‘Summer black’ grape under two-crop-a-year cultivation. J. Fruit Sci. 37, 226–234. doi: 10.13925/j.cnki.gsxb.20190312

